# From Knowledge to Action: Enhancing Herpes Zoster Vaccine Uptake in Diabetic Patients Through Targeted Interventions

**DOI:** 10.3390/vaccines14030209

**Published:** 2026-02-26

**Authors:** Marco Fabbri, Giorgio Cavalli, Valeria Frassineti, Paolo Di Bartolo, Chiara Reali, Elizabeth Bakken, Michela Morri, Gianluigi Belloli, Gianpiero Mancini, Manuela Chiavarini, Giulia Silvestrini

**Affiliations:** 1 Unit of Hygiene and Public Health Ravenna, Department of Public Health, Romagna Local Health Authority, 48121 Ravenna, Italy; 2 School of Hygiene and Preventive Medicine, University of Ferrara, 44121 Ferrara, Italy; 3 Diabetes Clinic of Ravenna, Romagna Local Health Authority, University of Bologna, 48121 Ravenna, Italy; 4 Unit of Hygiene and Public Health Forlì-Cesena, Department of Public Health, Romagna Local Health Authority, 47522 Cesena, Italy; 5 Unit of Hygiene and Public Health Rimini, Department of Public Health, Romagna Local Health Authority, 47624 Rimini, Italy; 6 Department of Public Health, Romagna Local Health Authority, 48121 Ravenna, Italy; 7 Department of Health Sciences, University of Florence, 50139 Florence, Italy

**Keywords:** herpes zoster, shingles, varicella zoster virus, vaccination, diabetes mellitus, risk groups, vaccine uptake, targeted interventions, active campaign

## Abstract

**Background/Objectives**: Vaccinations are a cost-effective strategy to reduce morbidity and mortality from infectious diseases, particularly in individuals with diabetes mellitus (DM). Despite recommendations for routine vaccination, uptake among adults with DM remains suboptimal. This study evaluates the effectiveness of interventions aimed at increasing Herpes Zoster (HZ) vaccine coverage in diabetic patients. **Methods**: This study included diabetic patients residing in the Romagna Local Health Authority (1.2 million inhabitants). Two complementary strategies were implemented: patient-oriented interventions (reminders/recalls) and provider-focused actions to engage general practitioners and diabetologists. Vaccination coverage in December 2023 was compared with December 2024, stratified by age, sex, and nationality. Primary outcomes were changes in coverage and intervention impact. Multivariate logistic regression assessed sociodemographic determinants of adherence. **Results**: As of 31 December 2023, 5182 diabetic patients had received at least one dose of Herpes Zoster vaccine (7.4% coverage). In 2024, overall coverage (11.1%) increased across all sociodemographic groups. Coverage rose to 12.8% in males and 9.0% in females and to 11.7% among Italian citizens, while foreign citizens showed lower absolute levels but a greater proportional increase. By age, individuals born after 1958 exhibited the largest relative growth, whereas those born in 1952–1958 reached the highest absolute coverage in 2024. Male sex, the 1952–1958 birth cohort, and Italian citizenship were associated with a higher likelihood of vaccination in both years. **Conclusions**: Our findings show a positive trend in Herpes Zoster vaccination among diabetic patients in Romagna HLA, driven by patient reminders, provider engagement, and age-targeted campaigns, while persistent sociodemographic disparities underscore the need for tailored strategies to optimize coverage.

## 1. Introduction

Vaccination strategies are globally acknowledged as some of the most effective, feasible, and economically advantageous public health interventions for mitigating the incidence, severity, and mortality associated with infectious diseases [1]. The role of vaccinations is crucial in individuals with chronic conditions such as diabetes mellitus, who are predisposed to a higher risk of infections due to alterations in innate and adaptive immune responses, chronic low-grade inflammation, vascular complications, and overall metabolic imbalance. As a result of this immune dysfunction and metabolic dysregulation, individuals with diabetes are not only more likely to contract infections but also to develop more severe clinical outcomes. This heightened vulnerability reflects the complex and bidirectional relationship between diabetes and infections, in which infections can destabilize glycemic control, while chronic hyperglycemia further impairs immune responses and increases susceptibility. Patients with diabetes also exhibit increased vulnerability to a range of vaccine-preventable diseases, including herpes zoster, for which diabetes represents one of the primary risk factors [2].

Herpes zoster results from the reactivation of latent varicella zoster virus (VZV) in the sensory ganglia, leading to viral replication and spread along sensory nerves. This process produces the typical painful or pruritic, blistering rash. The most frequent and burdensome complication is post-herpetic neuralgia (PHN), characterized by persistent pain after rash resolution, which may last for years and markedly impair quality of life [3]. HZ is also associated with higher risk of major cardiac and cerebrovascular events, possibly due to both the presence of varicella zoster virus in intracerebral arteries after the acute infection and the associated inflammation leading to cytokine release and endothelial dysfunction, the risk of stroke appears to be higher especially shortly after a HZ episode [4,5].

The magnitude of the risk of developing HZ in diabetic patients is conditioned by age, with the highest risk in individuals older than 65, and by presence of comorbidities [6].

PHN appears to be more severe and persistent in diabetic patients, with a higher risk for hospitalization, resulting in a significant cost increase in national healthcare and social systems [7,8].

As HZ seems to be related to higher health-related resources consumption by diabetic patient, especially after a HZ episode, immunization may not only serve as a critical measure to reduce the frequency and severity of these infections but may also substantially lower hospitalization rates, intensive care admissions, and infection-related mortality within this population [9,10].

Furthermore, vaccination reduces healthcare-associated costs and resource utilization, thereby representing a cost-effective component of integrated diabetes management programs [11].

Vaccination against herpes zoster in adults and in people with chronic conditions represents a crucial public health strategy to improve quality of life and to reduce both the costs and complications associated with the disease, such as postherpetic neuralgia. It is therefore urgent to promote harmonized immunization policies that encompass both older adults and at-risk groups, ensuring adequate access and reimbursement across European Union countries [12].

Current Italian guidelines highlight the importance of routine vaccinations as an integral part of diabetes care, tailored to mitigate infection risks in this vulnerable population [13]. The specific vaccination recommendations for diabetic population were adopted and integrated at the regional level in Emilia-Romagna through the Regional Vaccination Plan, including vaccinations against Herpes Zoster which is both recommended in type 1 and type 2 diabetic patient [14]. As such, routine immunization is strongly recommended for this group. However, vaccination coverage remains below the recommended threshold due to various barriers, including vaccine hesitancy, limited awareness, and inequitable access to healthcare services [15]. This persistent gap in coverage has prompted research into effective strategies to improve vaccine uptake. A recent systematic review and meta-analysis examined various approaches aimed at increasing vaccination coverage [16,17].

To close these immunization gaps, coordinated, multidisciplinary strategies are needed, integrating vaccination into routine diabetes care. Such approaches align with the WHO’s Immunization Agenda 2030 and are essential to reducing preventable disease burden in this high-risk population [11].

It has long been established that strategies involving patient reminders and provider prompts lead to a significant increase in vaccination rates compared to standard care [18]. In particular, healthcare providers play a crucial role in patients’ decision-making processes, with provider recommendation recognized as one of the strongest predictors of vaccine acceptance [19].

This study was designed to evaluate the effectiveness of targeted interventions intended to enhance Herpes Zoster vaccine uptake among individuals with diabetes, a population at heightened risk for vaccine-preventable diseases.

## 2. Materials and Methods

### 2.1. Study Population

The study population consisted of all individuals with a diagnosis of diabetes mellitus residing in the Romagna Local Health Authority (LHA). Diabetic patients were identified and extracted from the general resident population using the algorithm described below, applied to the LHA Romagna health information system. The underlying source population included all residents of the Romagna LHA, for whom general demographic characteristics and vaccination coverage data for the same study period were available (See Supplementary Material: Table S1—General Population Profile and Herpes Zoster Vaccine Uptake in the Romagna LHA, 2023–2024). These data describe the overall population—comprising healthy individuals, persons with diabetes, and individuals with other chronic conditions for which HZ vaccination is recommended—and provide the contextual framework within which the diabetic subgroup was defined.

### 2.2. Data Source

The algorithm integrates multiple routinely collected administrative data sources (e.g., hospital discharge records, exemption registries, drug dispensing archives, and outpatient care databases) to ensure a comprehensive and validated identification of diabetic cases. The algorithm works as follows:

Patients 18 years of age and older were selected upon their presence in at least one of the following databases:

1. Patients discharged from hospitals with a primary or secondary diagnosis of diabetes (ICD-9-CM code 250* or 648.0*) in the year 2023 or in the three previous years (source: national hospital discharge database).
2. Prescriptions of antidiabetic drugs (ATC code: A10A* and A10B*) prescribed in the year 2023 or in the three previous years (source: national database of pharmaceutical prescriptions). Only people who had at least two prescriptions of antidiabetic drugs at two different times were included.
3. The third source was the set of files of all subjects who obtained exemption from payment of drugs or laboratory testing due to a diagnosis of diabetes mellitus, both type 1 and type 2 diabetes, in the year 2023 or in the 3 previous years (source: regional registers of exemptions).

Herpes Zoster vaccination data were retrieved from the Public Health Unit’s vaccination registry, which records all dispensed vaccines.

### 2.3. Intervention

Two main types of interventions were implemented.

1. Patient-oriented interventions included a reminder, whereby all diabetic patients attending diabetes clinics were advised by their diabetologist to receive vaccination, and a recall, consisting of phone calls made by public health vaccination centers personnel to patients identified from lists of those not yet vaccinated to schedule appointments. Before proceeding with the phone-calls, verbal consensus was requested from each individual attending the diabetes clinics by the diabetologist.
2. Provider-focused interventions comprised educational sessions for GPs and diabetologists, distribution of information sheets on the benefits of vaccination, and the inclusion of vaccination coverage among diabetic patients as a budgetary objective of the Romagna LHA, functioning as a performance incentive.

The algorithm integrates multiple routinely collected administrative data sources (e.g., hospital discharge records, exemption registries, drug dispensing archives, and outpatient care databases) to ensure a comprehensive and validated identification of diabetic cases.

The implementation of this intervention included the delivery of a one-time recall personalized SMS message to the target cohort not yet vaccinated, claims through video showing diabetologist promoting the vaccination that were projected in waiting areas throughout the LHA sites, as well as articles published on the diabetic patient association’s local magazine. GPs were also targeted through the distribution of awareness letters.

In 2024, within the Romagna LHA, Herpes Zoster vaccination was primarily delivered through public health vaccination centers. Additionally, as part of a pilot project, GPs were authorized to administer the vaccine, with each GP allowed to vaccinate up to 15 patients.

During the study period, two vaccines against HZ were available: the live attenuated vaccine (VZL) and the recombinant zoster vaccine (RZV). As RZV provides superior and longer-lasting protection, it has consistently been offered free of charge in Emilia-Romagna to individuals with risk factors, such as diabetes, in accordance with guidelines [20,21].

### 2.4. Outcome

In order to answer the primary research question, which concerned the improvement of vaccination coverage, the selected outcome measures were overall vaccination coverage with at least one dose of HZV, as well as coverage at the district level and stratified by age, sex, and citizenship.

As a secondary outcome, in order to assess the impact of the different strategies implemented, a comparison was carried out by NHS facility of vaccine administration, evaluating the contribution of the Public Health Service and GPs, both overall and at the district level.

### 2.5. Statistical Analysis

First, a descriptive analysis of the study population was conducted. Absolute and relative frequencies were calculated for the main sociodemographic variables of diabetic patients residing in the Romagna LHA, including age, sex, and citizenship.

Subsequently, Herpes Zoster (HZ) vaccination coverage was assessed among the diabetic cohort. Coverage estimates for 2023 and 2024 were compared, and stratified analyses were performed to describe the distribution of vaccine uptake by age group, sex, and citizenship. Temporal comparisons were carried out to evaluate changes in coverage following the implementation of the targeted vaccination interventions.

Finally, multivariable logistic regression models were constructed to examine the association between sociodemographic characteristics and HZ vaccine uptake, as well as to evaluate the effect of the intervention on vaccination likelihood. Adjusted odds ratios (ORs) and their corresponding 95% confidence intervals (CIs) were calculated. All hypothesis tests were two-tailed, and statistical significance was set at *p* < 0.05. Data analysis and processing were performed using Stata 18.

## 3. Results

### 3.1. Demographics

Our study population comprised 70,465 individuals. Overall, 45.8% were female (n = 32,290) and 54.2% were male (n = 38,175). With respect to age, 31.3% were born after 1958 (n = 22,046), 18.2% were born between 1952 and 1958 (n = 12,846), and 50.5% were born before 1952 (n = 35,573). Regarding citizenship, the vast majority were Italian nationals (92.6%, n = 65,218), while 7.4% (n = 5247) were foreign citizens (Table 1).

### 3.2. Overall Coverage and Differences Across Population

As of 31 December 2023, 5182 diabetic patients of the study population had received at least one dose of Herpes Zoster vaccination, corresponding to an overall coverage of 7.4%.

Coverage rose significantly in 2024 versus 2023, with notable variation by sex, age, and citizenship. Coverage reached 12.8% in males and 9.0% in females, corresponding to relative year-over-year increases of 51.8% and 49.2%, respectively. This pattern suggests an improvement in coverage in both sexes, although the absolute coverage remained higher among males, in line with previously reported sex-related disparities in preventive health uptake.

Among Italian citizens, vaccine coverage increased from 7.8% in 2023 to 11.7% in 2024, reflecting a 50.5% rise. In contrast, foreign citizens showed lower absolute coverage (from 2.3% to 3.6%), but a higher relative year-over-year increase of 61.0%.

By age, individuals born after 1958 showed the largest relative change observed across all strata with an increase in coverage from 2.1% to 5.6%, corresponding to a 164.7% year-over-year increase, while those born between 1952 and 1958 (65–71 years) achieved the highest absolute coverage in 2024 (28.2%), with a 79.4% increase compared to 2023. Finally, the patients born before 1952 demonstrated more modest growth, from 4.7% in 2023 to 8.3% in 2024 (18.1% year-over-year increase).

### 3.3. Logistic Regression

A multivariate logistic regression model was created to evaluate the impact of sociodemographic determinants on adherence to vaccination recommendations (Table 2).

Male participants showed higher odds of adherence compared to females in both years, with an increase from OR = 1.28 (95% CI: 1.20–1.36) in 2023 to OR = 1.36 (95% CI: 1.30–1.43) in 2024.

Individuals born after 1958 exhibited markedly lower odds of adherence relative to the 1952–1958 cohort, although the odds ratio increased from 0.07 (95% CI: 0.07–0.08) in 2023 to 0.16 (95% CI: 0.15–0.17) in 2024.

Conversely, participants born before 1952 demonstrated odds ratios of 0.15 (95% CI: 0.14–0.16) in 2023 and 0.23 (95% CI: 0.22–0.24) in 2024.

Foreign participants consistently exhibited lower odds of adherence compared to Italian citizens, with stable estimates across years (OR = 0.30; 95% CI: 0.25–0.36 in 2023 and OR = 0.30; 95% CI: 0.26–0.35 in 2024).

## 4. Discussion

### 4.1. Main Findings

Our study showed an increase in herpes zoster vaccination coverage among diabetic patients in the Romagna LHA, from 7.4% in 2023 to 11.1% in 2024. Although absolute levels remain suboptimal, this rise of nearly four percentage points confirms that targeted strategies can positively impact vaccination uptake in this high-risk population.

### 4.2. Demographic Differences in Vaccination Uptake

Our findings underscore a positive trajectory in vaccine uptake. Although comparing 2023 to 2024 we recorded a 50.8% year over year increase in the HZ coverage in diabetic population resident in the Romagna LHA, we observed heterogeneity across demographic subgroups. The % year over year increase in coverage was higher in males (51.8% vs. 49.2%), in foreign citizens (61.0 vs. 50.5), and largely differed across age groups with the highest increase in those born after 1958 (164.7% vs. 79.4 in Born 1952–1958 vs. 18.1 in born before 1952).

Males were more likely to be vaccinated than females, and this finding is in line with evidence on vaccination behavior. Multiple studies report that women express lower intention to vaccinate compared to men [22,23,24,25,26].

Gender-related barriers, such as increased unpaid care responsibilities, restricted mobility to health services, and limited decision-making autonomy among women, have been identified as significant determinants of vaccination uptake. These factors can negatively influence access to vaccine services and health information, contributing to differential vaccine coverage by gender [27,28].

Studies also indicate that dimension of vaccine hesitancy differs between men and women, with women often reporting greater concerns about vaccine safety and health risks, possibly mediating gender differences in vaccination intentions and acceptance [22,29].

Despite foreign citizens being markedly less likely to be vaccinated compared with Italian citizens, the observed year-over-year increase suggests that our multi-level intervention may have also reached foreigners.

Finally, the highest coverage was observed in the age group born between 1952 and 1958, likely reflecting the combined effect of interventions targeted at diabetic patients and the regional policy offering free vaccination at age 65.

### 4.3. Interpretation

The extraction of the diabetic population from this larger administrative cohort allowed for a consistent assessment of vaccination uptake in a clinically relevant subgroup while maintaining comparability with coverage estimates in the general population.

The most marked rise was observed among individuals born after 1958, whose uptake more than doubled from 2.1% in 2023 to 5.6% in 2024. We can assume that the increase in vaccination coverage recorded among younger patients was primary driven by targeted activities for frail individuals (diabetic patients-oriented and provider-focused), rather than by age-based, although in 2024 the regional age-targeted campaign may have partially contributed to the increase in vaccination coverage in the population born in 1959.

Concerning the diabetic population born between 1952 and 1958, the year over year increase reflects the combined effect of the diabetes-focused patient intervention and the regional age-targeted campaign.

For individuals born before 1952, the observed increase is largely driven by the intervention targeting diabetic patients. Nevertheless, it should be considered that the prevalence of comorbid conditions conferring eligibility for vaccination rises substantially with age. As a result, within the general population, a considerable proportion of older adults is expected to present medical conditions for which vaccination is recommended by physicians in multiple settings.

These results suggest that structured interventions can increase vaccination coverage, particularly when strategies operate at multiple levels. In line with Jacobson Vann et al., reminder/recall systems have consistently been shown to increase immunization uptake, and also in our setting this intervention appears to have contributed to improved coverage [18]. Moreover, a Multi-Center Retrospective Observational Study conducted in the Romagna LHA recently evaluated the effect of Catch-Up campaign and recall activities for HZ vaccination. Although not specifically addressed in this study, geographical and cultural contexts could act as the turning points to deliver effective and efficient strategies aiming at reducing inequities and improve coverage in vulnerable populations [30].

Provider-focused strategies, particularly when multi-component, have proven effective in strengthening vaccination practices. Patient reminders, provider engagement, and population-based policies appear to act synergistically [31,32,33,34]. Notably, interventions delivered at the regional or national level—such as the routine offer of herpes zoster vaccination to those turning 65—seem especially effective in achieving measurable gains. Although not specifically targeted at diabetic patients, this measure likely contributed to the higher coverage observed in the population born between 1952 and 1958.

Our study has several strengths, including the large population-based cohort encompassing the entire resident diabetic population from the Romagna Local Health Authority. This approach minimizes the risk of selection bias and enhances the generalizability of the findings to comparable healthcare settings. Similar population-based designs have been widely used in vaccination uptake research. Moreover, diabetic individuals were identified through a robust administrative algorithm integrating multiple routinely collected data sources—including hospital discharge records, pharmaceutical dispensing archives, exemption registries, and outpatient care databases—ensuring a high level of case completeness. Algorithms of this type are well-established in epidemiological surveillance and have demonstrated good validity in previous studies [35]. Importantly, the comparative analysis across two consecutive years and the multivariable adjustment allows for the comparison of temporal changes in uptake. The use of a multivariable analytical approach further strengthens the internal validity of the findings by accounting for key confounders.

Lastly, the assessment of Herpes Zoster vaccination uptake within the context of routine clinical and organizational practice, provides insights directly applicable to the operational management of immunization programs. Real-world evidence is increasingly recognized as essential for informing vaccination strategies in chronic disease populations.

However, some limitations must be acknowledged: Diabetic patients were identified using the 2023 administrative algorithm rather than an updated 2024 version. Although the algorithm is validated, as with all studies relying on routinely collected data, some degree of misclassification of both diabetes status and vaccination records cannot be excluded. However, such misclassification is expected to be non-differential and therefore unlikely to fully account for the observed trends.

Despite multivariable adjustment, unmeasured factors—such as socioeconomic status, health literacy, cultural barriers, or attitudes toward vaccination—may have influenced uptake. Residual confounding is a well-recognized limitation of observational studies relying on administrative datasets.

Another limitation of this study is the relatively short observation window, covering a two-year period, which limits the ability to assess longer-term trends or the persistence of intervention effects over time. Longer follow-up would be required to evaluate sustained changes in vaccination behavior.

Although the study evaluates targeted interventions, it does not directly measure their intensity, quality, or heterogeneity in implementation. In the cohort born between 1952 and 1958, it is not possible to determine precisely which intervention each individual received.

Finally, our study did not specifically address cost-effectiveness of patient-oriented and provider-focused interventions. As patient-oriented intervention such as Digital health interventions (DHIs), such us SMS or videos, have been used in various vaccination programs and are known to be a cost effective measure used to increase vaccination coverage, further studies should focus on the evaluation of provider-focused intervention specifically targeting HZV in the diabetic population [36].

1. Patient-oriented interventions included a reminder, whereby all diabetic patients attending diabetes clinics were advised by their diabetologist to receive vaccination, and a recall, consisting of phone calls made by public health vaccination centers personnel to patients identified from lists of those not yet vaccinated to schedule appointments. Before proceeding with the phone-calls, verbal consensus was requested from each individual attending the diabetes clinics by the diabetologist.
2. Provider-focused interventions comprised educational sessions for GPs and diabetologists, distribution of information sheets on the benefits of vaccination, and the inclusion of vaccination coverage among diabetic patients as a budgetary objective of the Romagna LHA, functioning as a performance incentive.

## 5. Conclusions

In conclusion, our findings highlight the need to reinforce vaccination interventions, ensuring that they are systematically integrated into care pathways. While a direct causal relationship cannot be established, the increase in coverage plausibly reflected the combined impact of these interventions, consistent with evidence from the literature. Since our study focused on available data from 2023 and 2024 to assess long-term effects, it would be useful to monitor HZV coverage rates in the diabetic population in the following years. Dedicated strategies addressing gender disparities and the lower uptake among foreign citizens are warranted, alongside continued investment in structured, multi-level approaches. Such efforts are essential to reduce inequities and further improve coverage in vulnerable populations.

## Figures and Tables

**Table 1:** Sociodemographic characteristics and Herpes Zoster vaccine uptake among diabetic patients identified by the administrative algorithm, Romagna LHA (2023–2024). Coverage indicates the percentage of diabetic population vaccinated with at least one dose of HZV, either VZL or RZV.

	% 2023 (N)	% Coverage 2023 (N)	% Coverage 2024 (N)	% Increase (N)	% Year over Year Increase
Sex (*)					
Female	45.8 (32,290)	6.1 (1955)	9 (2916)	3 (961)	49.2
Male	54.2 (38,175)	8.5 (3227)	12.8 (4897)	4.4 (1670)	51.8
Age (*)					
Born after 1958	31.3 (22,046)	2.1 (464)	5.6 (1228)	3.5 (764)	164.7
Born 1952–1958	18.2 (12,846)	23.9 (3065)	28.2 (3620)	4.3 (555)	79.4
Born before 1952	50.5 (35,573)	4.7 (1653)	8.3 (2965)	3.7 (1312)	18.1
Citizenship (*)					
Italian	92.6 (65,218)	7.8 (5064)	11.7 (7623)	3.9 (2559)	50.5
Foreign	7.4 (5247)	2.3 (118)	3.6 (190)	1.4 (72)	61.0
Total	100 (70,465)	7.4 (5182)	11.1 (7813)	3.7 (2631)	50.8

(*) Chi square *p* > 0.001.

**Table 2:** Multivariable logistic regression evaluating temporal changes in determinants and equity of Herpes Zoster vaccine uptake in diabetic patients (2023 vs. 2024).

	Odds Ratio 2023	(IC 95%)	Odds Ratio 2024	(IC 95%)
Sex				
Female	1	-	1	-
Male	1.28	1.20–1.36	1.36	1.30–1.43
Age				
Born 1952–1958	1	-	1	-
Born after 1958	0.07	0.07–0.08	0.16	0.15–0.17
Born before 1952	0.15	0.14–0.16	0.23	0.22–0.24
Citizenship				
Italian	1	-	1	-
Foreign	0.30	0.25–0.36	0.30	0.26–0.35

## Data Availability

The data presented in this study are available on request from the corresponding author.

## Supplementary Materials

The following supporting information can be downloaded at: https://www.mdpi.com/article/10.3390/vaccines14030209/s1, Table S1. General Population Profile and Herpes Zoster Vaccine Uptake in the Romagna LHA, 2023–2024.
