# Supplementary material for: From Knowledge to Action: Enhancing Herpes Zoster Vaccine Uptake in Diabetic Patients Through Targeted Interventions
Source: Vaccines (Basel). 2026 Feb 26;14(3):209. doi: 10.3390/vaccines14030209 (PMC13030708; doi:10.3390/vaccines14030209)
Supplement: Supplementary file 1 [file vaccines-14-00209-s001.zip › vaccines-4113019-supplementary.pdf]

Table S1. General Population Profile and Herpes Zoster Vaccine Uptake in the Romagna LHA, 2023-2024.

		(N) general population HLA Romagna 2023	% (N) general population HLA Romagna 2023	% (N) HZ Coverage in general population HLA Romagna 2024	% (N) HZ Coverage in general population HLA Romagna 2024
Sex (*)	Female	484665	51,53	2,99	4,00
	Male	455809	48,47	3,10	4,27
Age (*)	Born after 1958	678674	72,16	0,24	0,83
	Born 1952-1958	92360	9,82	23,02	26,16
	Born before 1952	169440	18,02	3,37	5,33
Citizenship (*)	Italian	n.a.	n.a.	n.a.	n.a.
	Foreign	n.a.	n.a.	n.a.	n.a.
Total		940474	100,00	3,04	4,13
